# Statistical optimization, kinetic, equilibrium isotherm and thermodynamic studies of copper biosorption onto *Rosa damascena* leaves as a low-cost biosorbent

**DOI:** 10.1038/s41598-022-12233-1

**Published:** 2022-05-20

**Authors:** Mustafa A. Fawzy, Hatim M. Al-Yasi, Tarek M. Galal, Reham Z. Hamza, Tharwat G. Abdelkader, Esmat F. Ali, Sedky H. A. Hassan

**Affiliations:** 1 Department of Biology, College of Science, Taif University, P.O. Box 11099, Taif, 21944 Saudi Arabia; 2 Department of Biology, College of Science, Sultan Qaboos University, Muscat, 123 Oman; 3 Department of Botany and Microbiology, Faculty of Science, New Valley University, El-Kharga, 72511 Egypt

**Keywords:** Biotechnology, Plant sciences, Environmental sciences

## Abstract

In this study, *Rosa damascena* leaf powder was evaluated as a biosorbent for the removal of copper from aqueous solutions. Process variables such as the biosorbent dose, pH, and initial copper concentration were optimized using response surface methodology. A quadratic model was established to relate the factors to the response based on the Box–Behnken design. Analysis of variance (ANOVA) was used to assess the experimental data, and multiple regression analysis was used to fit it to a second-order polynomial equation. A biosorbent dose of 4.0 g/L, pH of 5.5, and initial copper concentration of 55 mg/L were determined to be the best conditions for copper removal. The removal of Cu^2+^ ions was 88.7% under these optimal conditions, indicating that the experimental data and model predictions were in good agreement. The biosorption data were well fitted to the pseudo-second-order and Elovich kinetic models. The combination of film and intra-particle diffusion was found to influence Cu^2+^ biosorption. The Langmuir and Dubinin–Radushkevich isotherm models best fit the experimental data, showing a monolayer isotherm with a *q*_*max*_ value of 25.13 mg/g obtained under optimal conditions. The thermodynamic parameters showed the spontaneity, feasibility and endothermic nature of adsorption. Scanning electron microscopy, energy-dispersive X-ray spectroscopy, and Fourier transform infrared spectroscopy were used to characterize the biosorbent before and after Cu^2+^ biosorption, revealing its outstanding structural characteristics and high surface functional group availability. In addition, immobilized *R. damascena* leaves adsorbed 90.7% of the copper from aqueous solution, which is more than the amount adsorbed by the free biosorbent (85.3%). The main mechanism of interaction between *R. damascena* biomass and Cu^2+^ ions is controlled by both ion exchange and hydrogen bond formation. It can be concluded that *R. damascena* can be employed as a low-cost biosorbent to remove heavy metals from aqueous solutions.

## Introduction

With the development of agriculture, industrial activities, and other human activities, various heavy metals have been released into water, and water resources have been severely contaminated^1^. Heavy metal pollution has been a commonly researched topic for a long time because heavy metals are difficult to remove and are highly toxic^2^. Copper and its composites are the most frequent heavy metal contaminants in the environment, according to the Environmental Protection Agency^3^. Wastewater from mining companies, tanneries, metal plating plants and refineries is the most prevalent source of copper^4^.

Copper is a trace metal that numerous enzymes require for catalysis in living organisms. High quantities of copper, however, may be highly poisonous and cause major health concerns. Ingestion of high levels of copper causes the copper to accumulate in the liver, which can lead to anemia, gastrointestinal issues, and renal difficulties^5^. As a result, research into low-cost alternatives to presently available processes has been required by the need for safe, more effective and less expensive approaches for removing copper ions from wastewater. Several strategies for the removal of heavy metals from wastewaters have been developed in recent years. Some of these approaches include solvent extraction, membrane filtration, chemical reduction and precipitation, coagulation and ion exchange. The primary drawbacks of these procedures include the need for significant amounts of chemicals, residual metal solubility, costly capital investment, high operating costs, and the formation of a large quantity of sludge^6^. Adsorption is a potential approach to processing and treating wastewater due to its cost and efficiency^7^. Recent research has shown that a variety of typical agricultural waste products, microorganisms, algae, and natural polymers are excellent biosorbents for heavy metal removal^8–10^. Different parts of plants and their wastes are available and are more affordable than chemically modified products. As a result, the majority of adsorption research has focused on untreated plant products and wastes^11,12^.

Roses are shrubs that are commonly cultivated worldwide. These decorative plants are also utilized in medicine, fragrances, and scented products. Rose water, rose oil, and rose waste biomass are the three major products of rose distillation^13^. Rose flower biomass has been examined as a biosorbent after distillation in a number of studies^13–16^.

However, to the best of our knowledge, rose leaves have not been reported for use as waste biomass for the biosorption of copper ions. Therefore, in this study, *Rosa damascena* leaf powder was employed as a cost effective biosorbent to examine its efficiency in removing copper ions from aqueous solutions. A response surface methodology (RSM) was used to optimize the process variables and to analyze the influences of different factors on the examined response, providing significant advantages, as this methodology saved time, effort, and resources. In addition, RSM is more applicable since it can be used to predict and evaluate the interactive influences of different factors and depict their influence on a process^17–19^. Three independent factors, including biosorbent dose, pH, and initial Cu^2+^ concentration, were optimized to maximize copper removal by *Rosa damascena* leaf powder. Furthermore, we assessed the efficiency of immobilized *Rosa damascena* biomass as a Cu^2+^ ion biosorbent. Kinetic, equilibrium, and thermodynamic investigations were carried out to study the nature of the biosorbent and the biosorption process. The biosorbent was characterized by SEM, EDX and FTIR.

## Results and discussion

### Effect of metal concentration

**Figure 1:**
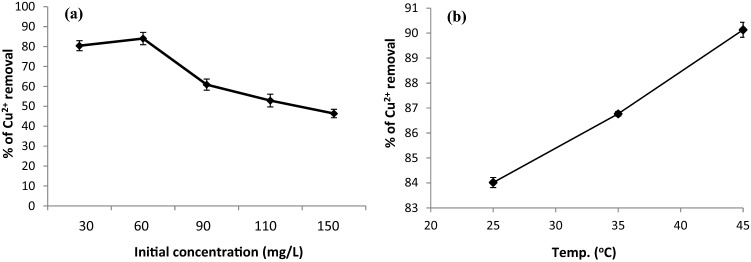
Effect of (**a**) Initial Cu^2+^ concentration, and (**b**) Temperature on the biosorption of Cu^2+^ ions onto *R. damascena* leaves. Data is presented as average ± SE of three replicates.

The initial metal concentration is a critical factor that influences the driving force of biosorption systems^17^. The data in Fig. 1a depict the influence of different metal concentrations (30–150 mg/L) on Cu^2+^ biosorption of onto the *R. damascena* biomass. The data showed that as the concentrations of Cu^2+^ increased from 60 to 150 mg/L, the copper removal declined from 84.0 to 46.3% (Fig. 1a) because at low Cu^2+^ concentrations, all active sites on the surface of *R. damascena* are vacant, but at high concentrations of Cu^2+^ ions, the number of binding sites is restricted. These binding sites are filled quickly, resulting in a significant reduction in Cu^2+^ biosorption. Several researchers have reported similar findings^20,21^.

### Effect of temperature

The influence of temperature on Cu^2+^ ion removal by *R. damascena* leaf biomass was studied at three different temperatures (25, 35, and 45 °C; Fig. 1b). The data show that the removal of Cu^2+^ ions increased from 84.0 to 90.1% as the temperature was increased, showing that the biosorption process is endothermic. The highest removal of Cu^2+^ (90.1%) occurred at a high temperature (45 °C), which may be regarded as optimal for Cu^2+^ biosorption. Increases in adsorbate affinity for the surface of adsorbent, increases in adsorbent pore size, and increases in driving force to overcome the mass transfer resistance of the adsorbate between the adsorbent surface and aqueous solution may all contribute to the increased biosorption at higher temperatures^22^.

### Box–Behnken experimental design

A Box–Behnken design (BBD) was utilized to decrease the number of tests and predict the best conditions for copper removal by *R. damascena* leaf powder. Seventeen experiments were carried out using a BBD with varied combinations of three parameters at three levels to maximize the removal of Cu^2+^ ions from aqueous solution. Table 1 illustrates the actual and predicted removal percentages of Cu^2+^ for the 17 runs of the design matrix. The results showed that the copper removal varied greatly depending on the independent parameters. The copper removal by *R. damascena* leaves varied between 44.25 and 87.68%. In run 4, with a biosorbent dose of 5 g/L, pH of 6, and initial Cu^2+^ concentration of 60 mg/L, the maximum copper removal was obtained with a value of 87.68%. Based on the Box–Behnken design results, a second-order polynomial equation was created to characterize the relationship between the independent parameters and the response, and the final model generated by backward elimination of insignificant parameters is as follows (Eq. (1)):

1 $${\text{Cu}}^{{{2} + }} \;{\text{removal percentage }} = { 57}.{2} + { 1}0.{54}A + { 5}.{13}B - { 4}.{28}C + { 5}.{71}A^{2} ,$$

where *A*, *B* and *C* are the biosorbent dose, pH and initial concentration of copper ions, respectively.

**Table 1:** Box–Behnken design factors with coded and un-coded factors and data of the experimental response.

Run	Factor 1 A: biosorbent dose (g/L)	Factor 2 B: pH	Factor 3 C: initial Cu^2+^ conc. (mg/L)	Response: Cu^2+^ removal (%)	Predicted values
1	1 (− 1)	2 (− 1)	60 (0)	44.25	47.28
2	5 (+ 1)	2 (− 1)	60 (0)	64.03	68.36
3	1 (− 1)	6 (+ 1)	60 (0)	51.67	57.53
4	5 (+ 1)	6 (+ 1)	60 (0)	87.68	78.62
5	1 (− 1)	4 (0)	30 (− 1)	56.80	57.22
6	5 (+ 1)	4 (0)	30 (− 1)	77.90	78.31
7	1 (− 1)	4 (0)	90 (+ 1)	56.90	47.58
8	5 (+ 1)	4 (0)	90 (+ 1)	64.34	68.67
9	3 (0)	2 (− 1)	30 (− 1)	62.97	56.93
10	3 (0)	6 (+ 1)	30 (− 1)	68.43	67.18
11	3 (0)	2 (− 1)	90 (+ 1)	50.91	47.29
12	3 (0)	6 (+ 1)	90 (+ 1)	55.39	57.54
13–17*	3 (0)	4 (0)	60 (0)	60.20	57.24

*Mean value of five center point assays.

Table 2 summarizes the results of the analysis of variance (ANOVA) of the established model. The model was highly statistically significant, as evidenced by the high *F* value (10.26) and low *p* value (˂ 0.001). Furthermore, the *F* value of the lack of fit was 1.69, suggesting that the lack of fit is not significant (as the *p* value is larger than 0.05) in comparison to the true error, so the model validity may be affirmed^23^.

**Table 2:** Analysis of variance (ANOVA) of the response surface quadratic model of the biosorption of Cu^2+^ ions onto *R. damascena*.

Source	*SS*	*df*	*MS*	*F* value	*p* value Prob > *F*
Model	1423.4	4	355.8	10.26	0.0008*
Residual	416.4	12	34.70	–	–
Lack of fit	321.4	8	40.18	1.69	0.321**
Pure error	95.0	4	23.74	–	–
Correlation total	1839.8	16	–	–	–
R^2^ = 0.87	Adj. R^2^ = 0.80	Pred. R^2^ = 0.70	Adeq. precision = 9.8	CV% = 8.83	Mean = 59.92

*Df* degree of freedom, *SS* squares sum, *MS* mean sum of squares, *CV* Coefficient of variation.

*Significant at *p* ˂ 0.05.

**Not significant at *p* > 0.05.

The determination coefficient (*R*^2^) and adjusted *R*^2^ were used to assess the model’s fit. The *R*^2^ values ranged between 0 and 1.0, with values of approximately 1.0 indicating that the model is more accurate. However, under certain conditions, a larger *R*^2^ value indicates that the model has a large number of insignificant variables, which indicates a poor response. As a result, the adjusted *R*^2^ was developed, which adjusts the value of *R*^2^ based on the number of variables and sample size in the model. The high *R*^2^ value (0.87; Table 2) in this investigation implies that the actual and expected values are well correlated, and the model can explain 87.0% of the variability in the response. The adjusted *R*^2^ of 0.80 agrees well with the *R*^2^ value of 0.87, indicating that the model is valid. The actual and expected results were highly correlated, with the adjusted *R*^2^ value being high and close to the predicted *R*^2^ value (Table 2).

Moreover, the value of the variation coefficient as an estimate of the standard error was less than 10%, indicating that the model was reproducible^24^. The signal-to-noise ratio indicated adequate precision. In this investigation, a ratio of 9.8 (higher than 4) was found to be sufficient^25^. As a result, the model may be utilized to explore the design space. Therefore, biosorption studies may be conducted using this model.

### Effect of interactive variables

**Figure 2:**
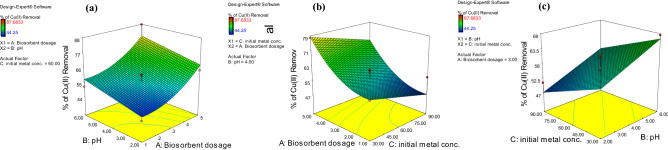
3-D response surface plots for Cu^2+^ removal showing the interaction influences of (**a**) biosorbent dose and pH, (**b**) biosorbent dose and initial copper concentration, and (**c**) pH and initial copper concentration.

In order to understand the impacts of the interactions of factors on the investigated response, 3-D response surface plots were made using the second-order Eq. (1) (Fig. 2). Each plot depicts the impact of two independent factors on the response within the examined ranges, while all other factors were held constant.

In Fig. 2a, 3-D plots depict the reciprocal interaction between the biosorbent dose and pH on Cu^2+^ removal by *R. damascena* leaf biomass. The data revealed that raising the biosorbent dose and pH improved Cu^2+^ biosorption by *R. damascena* leaves. The ANOVA findings also demonstrated that the biosorbent dose was significant and had a positive influence on the efficiency of copper removal (*p* = 0.0003; Table 3) in linear term. The number of active binding sites for the biosorption process is determined by the biosorbent dose^26^. As the biosorbent dose was increased, the number of binding sites on the surface of *R. damascena* leaves rises, resulting in a higher percentage of copper removal^26^. The elimination of copper by *R. damascena* leaves is also pH-dependent. The speciation of ions in aqueous solution and the dissociation state of biosorbent’s superficial functional groups are both affected by pH^17^. The pH exhibited a significant positive influence on the removal of copper by *R. damascena* leaves in linear term, according to the ANOVA results (Table 3). When the pH was raised from 2.0 to 6.0, the biosorption of Cu^2+^ increased. However, copper biosorption onto *R. damascena* leaves was minimal at lower pH. However, when the pH was 6.0, the maximum removal efficiency was observed because the surface charge of the biomass is positive at lower pH, which limits cation biosorption. Additionally, H^+^ ions compete with copper ions for active sites, leading to reduced biosorption. The competitive impact of H^+^ ions and electrostatic repulsions between cations and surface sites reduced as the pH was increased. As a result, metal biosorption also increased^27^. Fawzy^20^ stated that a pH of 5.0 was the most effective pH for copper removal by *Codium vermilara*.

**Table 3:** Analysis of variance (ANOVA) for the coefficients of the quadratic model of the biosorption of Cu^2+^ ions onto *R. damascena*.

Model term	*CE*	*df*	*SE*	*F* value	*p* value Prob > *F*
Intercept	57.24	1	1.96	–	–
A-Biosorbent dose	10.54	1	2.08	25.63	0.0003
B-pH	5.13	1	2.08	6.06	0.03
C-initial Cu^2+^ conc.	− 4.82	1	2.08	5.36	0.039
A^2^	5.71	1	2.86	3.98	0.069

*CE* coefficient estimate, *df* degree of freedom, *SE* standard error.

Figure 2b depicts the mutual impacts of the biosorbent dose and initial copper concentration on the effectiveness of copper removal by *R. damascena* leaves.

When the biosorbent dose was increased from 1 to 5 g/L, the copper removal efficiency increased. More binding sites on the surface of *R. damascena* leaves become available to the copper ions as the biosorbent dose increases, resulting in enhanced removal efficiency. At a biosorbent dose of 5 g/L, the optimal removal effectiveness of 79% could be achieved. Generally, a higher biosorbent dose and lower copper concentrations improved the biosorption process^28^.

As a result, raising the concentration of copper ions had a significant negative impact on Cu^2+^ ion removal (Table 3). Because more copper ions from the solution connected with the binding sites at lower copper concentrations, the biosorption of Cu^2+^ ions gradually increased; however, as the concentrations of copper were increased, biosorption was reduced due to biosorbent site saturation, and a large number of ions competed for the residual binding sites in the biosorbent.

The joint influence of pH and initial Cu^2+^ concentrations on metal ion removal was also investigated in the pH range of 2–6 and initial copper concentrations of 30–90 mg/L, as shown in Fig. 2c. The findings revealed that the removal of copper ions decreases as the pH is decreased. ANOVA revealed that the biosorbent dose was the most statistically significant factor that influenced the removal of copper (*p* = 0.0003), followed by pH (*p* = 0.03) and initial copper concentration (*p*˂ 0.05; Table 3).

### Validation of the optimized variables

The goal of the optimization was to optimize the independent parameters of Cu^2+^ ion elimination by *R. damascena* leaf powder. The aim was to optimize the copper removal efficiency to achieve the maximum rate of Cu^2+^ removal. The average Cu^2+^ removal efficiency was compared to the expected value through experiments conducted in triplicate under optimized conditions. With a biosorbent dose of 4.0 g/L, pH of 5.5, and initial copper concentration of 55 mg/L, the highest expected Cu^2+^ elimination by *R. damascena* biomass was achieved. The experimentally observed copper removal efficiency (88.7%) was found to be in accordance with the expected value (87.4%) calculated by the design expert software, implying that the optimized conditions were ideal.

### Effect of contact time and kinetic models

**Figure 3:**
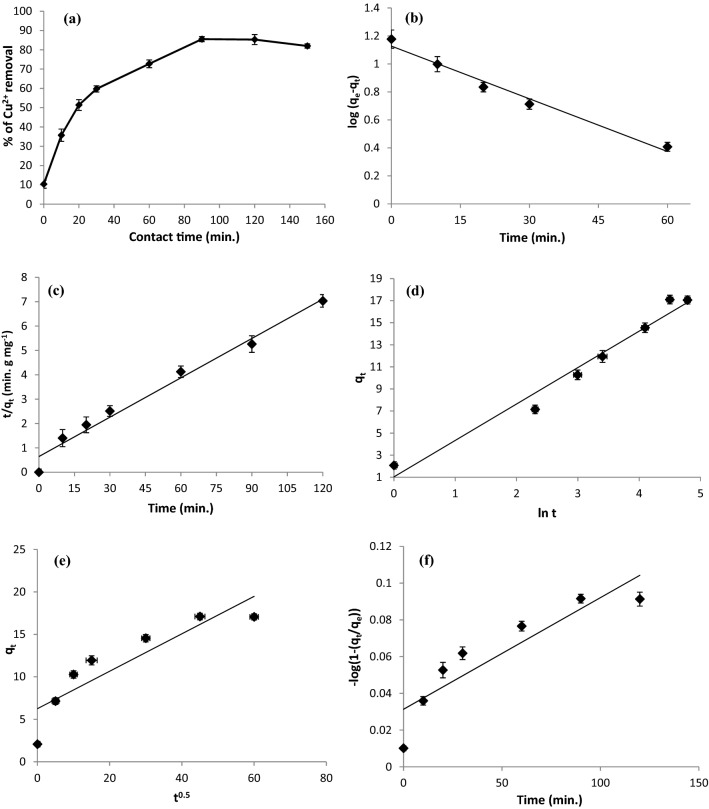
(**a**) Effect of contact time, (**b**) Pseudo-first-order plot, (**c**) Pseudo-second-order plot, (**d**) Elovich plot, (**e**) Intra-particle diffusion plot, and (**f**) film diffusion plot for the biosorption of Cu^2+^ ions onto *R. damascena* leaves. Data is presented as average ± SE of three replicates.

The impact of contact time on Cu^2+^ ion biosorption was used to evaluate the kinetics. Copper biosorption was examined under the optimal conditions of a 4.0 g/L biosorbent dose, pH 5.5, and an initial Cu^2+^ concentration of 55 mg/L by varying the biosorption time from 0 to 150 min (Fig. 3a). In the first 30 min, the rate of Cu^2+^ ion elimination was obviously fast. However, after equilibrium was reached, the biosorption efficiency increased until it was steady, and within 90 min, over 85.5% of the total metal was eliminated. The rates of adsorption and desorption were in dynamic equilibrium, and no additional biosorption was observed after this optimal equilibrium duration^29^. Because copper ions came into contact with unoccupied surface biosorption sites, the biosorption of copper was initially quicker; however, after adsorption proceeded at equilibrium for 90 min, the biosorption sites became saturated, and no further biosorption occurred^30^.

Various kinetic models can be used to explain the mechanism and rate of metal ion sorption^31^. The biosorption kinetics of copper ions on *R. damascena* leaf biomass was studied using the pseudo-first-order, pseudo-second-order, Elvoish, intra-particle and film diffusion models.

### Pseudo-first-order kinetic model

This model describes the adsorption of one adsorbate molecule onto one active site of the biosorbent. It is expressed as follows:

2 $$Log \left({q}_{e}-{q}_{t}\right)=Log {q}_{e}-\frac{{K}_{1} t}{2.303},$$

where *q*_*e*_ and *q*_*t*_ (mg/g) represent the quantity of copper ions absorbed by the biomass of *R. damascena* at equilibrium and at any time, respectively, and *K*_1_ (min^−1^) represents the rate constant of the pseudo-first-order model.

The constants *K*_1_ and *q*_*e*_ were estimated from the slope and intercept by plotting log (*q*_*e*_ − *q*_*t*_) vs. time, respectively (Fig. 3b).

The high value of the determination coefficient (*R*^2^ = 0.980) indicates that the experimental results accurately fit the pseudo-first-order model for describing copper ion biosorption kinetics. However, high *∆q*_*e*_ and *X*^2^ values (23.3 and 1.02, respectively; Table 4) suggested that the pseudo-first-order kinetic model does not exhibit good regression. In addition, the difference between the copper ion quantity biosorbed onto the *R. damascena* surface estimated by experiments (*q*_*e* exp_.; 17.1 mg/g) and the modeled value (*q*_*e* calc_.; 13.4 mg/g) were larger. This result suggests that the biosorption process involved both the copper ions and biosorbent^32^. Therefore, the pseudo-first-order model is unable to describe the experimental data of Cu^2+^ biosorption onto the *R. damascena* biomass. The sorption kinetics of various metal ions onto various adsorbents has been described with similar findings^15,33,34^.

**Table 4:** Kinetic model parameters of the biosorption of Cu^2+^ ions onto *R. damascena*.

Parameters		Values
Experimental data	*q*_*e*_ (exp.) (mg/g)	17.1
Pseudo-first order	*q*_*e*_ (cal.) (mg/g)	13.4
	*k*_1_ (min^−1^)	0.029
	*∆q*_*e*_	23.3
	*X*^2^	1.02
	*R*^2^	0.980
Pseudo-second order	*q*_*e*_ (cal.) (mg/g)	18.6
	*k*_2_ (g/mg min)	0.005
	*h* (g/mg min)	1.55
	*∆q*_*e*_	12.25
	*X*^2^	0.12
	*R*^2^	0.983
Elovich	*α* (g/mg min)	1.30
	*β* (g/mg)	0.303
	*∆q*_*e*_	3.53
	*X*^2^	0.192
	*R*^2^	0.971
Intra-particle diffusion	*K*_*i*_ (mg/g min^0.5^)	0.22
	*C*_*i*_ (mg/g)	6.25
	*R*^2^	0.801
Film diffusion	*D*_*f*_	0.001
	*R*^2^	0.821

The rate constant of pseudo-first-order (*K*_1_ = 0.029 min^−1^; Table 4) is not a quantifiable value that can clarify the rapid equilibrium of the biosorption of Cu^2+^ ions onto the *R. damascena* leaf surface reported within 30 min. As a result, this model was shown to be inadequate for accurately modeling copper biosorption by *R. damascena* biomass.

### Pseudo-second-order kinetic model

In the pseudo-second-order kinetic model, chemical adsorption, which includes the exchange or sharing of electrons between the adsorbate and the adsorbent, controls the process of adsorption. This model can be described as follows:

3 $$\frac{t}{{q}_{t}}= \frac{1}{{K}_{2}{q}_{e}^{2}}+\frac{t}{{q}_{e}},$$

where *K*_2_ is the pseudo-second-order rate constant (g/mg min), which may be used to calculate the initial rate of biosorption (*h*; g/mg min).

4 $$h={K}_{2}{q}_{e}^{2}.$$

The kinetic constants *K*_2_ and *q*_*e*_ were calculated from the intercept and slope of *t/q*_*t*_ against *t* plot, respectively (Fig. 3c).

The rate constant of pseudo-second-order (*K*_*2*_) and the initial rate of adsorption (*h*) were 0.005 and 1.55 g/mg min, respectively (Table 4). At the beginning of the biosorption process, the *h* value indicated the rapid biosorption of copper ions. The high determination coefficient (*R*^2^ = 0.983) and the low values of *∆q*_*e*_ and *X*^2^ (12.25 and 0.12, respectively; Table 4) indicated that the pseudo-second-order model best fit the experimental data for Cu^2+^ ion biosorption on the *R. damascena* leaf surface. Additionally, the experimental *q*_*e*_ (17.1 mg/g) was relatively similar to the calculated *q*_*e*_ (18.6 mg/g). Therefore, the pseudo-second-order model was chosen because it had the best fit, demonstrating significant interactions between the adsorbate and the adsorbent and indicating the occurrence of copper chemisorption on the surface of the *R. damascena* leaves. The results of several studies support the pseudo-second-order model for the adsorption of copper ions onto different biosorbents, including studies on the adsorption of Cu^2+^ onto activated rubber wood sawdust^35^, *Tectona grandis* leaf^36^, sour orange residue^37^, banana trunk fiber^38^, sulfur-modified bamboo powder^39^, and *Platanus orientalis* leaf powder^40^.

### Elovich model

The Elovich model is utilized to explain the kinetics of chemical adsorption of a gas onto solid adsorbents, but it has been proven to be effective in describing various types of adsorption. The Elovich model can be represented by the following equation:

5 $${q}_{t}=\frac{1}{\beta }\mathrm{ln}\left(\mathrm{\alpha \beta }\right)+\frac{1}{\beta }\mathrm{ln}\left(\mathrm{t}\right),$$

where *α* (mg/g min) represents the initial rate of sorption, and *β* (g/mg) is a constant that represents desorption.

To study the mechanism of Cu^2+^ biosorption, the experimental data were fitted to the Elovich kinetic model (Fig. 3d). A graph of *q*_*t*_ versus *lnt* was plotted, and the Elovich constants (*α* and *β*) were determined from the intercept and slope, respectively. The extent of chemisorption is proportional to the value of *α*. The high value of the Elovich constant (*α* = 1.3 g/mg min; Table 4) implies that chemisorption is the rate-limiting stage and biosorption proceeded via a pseudo-second-order mechanism.

The lower the value of the Elovich constant *β* is, the lower the chemisorption activation energy, implying that adsorption occurs quickly^41^. In the current investigation, the *β* value was fairly low (0.303 g/mg), suggesting a low activation energy of chemical adsorption. In addition, the high value of the determination coefficient (*R*^2^ = 0.971) and the low *∆q*_*e*_ and *X*^2^ values (3.53 and 0.192, respectively; Table 4) show that the experimental data fit the Elovich kinetic model well.

From the previous data, it can be concluded that, the *∆q*_*e*_ and *X*^2^ values were found to be smaller for the pseudo-second-order and Elovich kinetic models; with higher *R*^2^ values than to those of the pseudo-first-order. Thus, the pseudo-second-order and Elovich kinetic models are the best fit models for the biosorption of copper ion onto the *R. damascena* leaves.

### Intra-particle and film diffusion models

The mechanism of diffusion influencing the Cu^2+^ biosorption process was also evaluated by intra-particle and film diffusion models^42^.

The intra-particle diffusion kinetic model is related to adsorbate diffusion to the inner pores as the rate-controlling step, which is represented by Eq. (6):

6 $${q}_{t}= {K}_{i}{t}^{1/2 }+{C}_{i},$$

where *K*_*i*_ denotes the intra-particle diffusion rate constant (mg/g min^0.5^) and *C*_*i*_ denotes the intercept.

The film diffusion model is represented by the following Eq. (7):

7 $$-\mathrm{log }(1-\left(\frac{{q}_{t}}{{q}_{e}}\right)= {D}_{f}\cdot t,$$

where *D*_*f*_ is the film diffusion rate constant (min^-1^).

The trendline of the linear plot in Fig. 3e does not pass through the origin, proposing that intra-particle diffusion is not the sole rate-limiting stage of biosorption. Figure 3e also exhibited the multilinearity of the plot, which has two sections. The first section shows that Cu^2+^ ions are transferred from the solution to the external surface of the *R. damascena* biomass via film diffusion. Moreover, the film diffusion plot of − log (1 − (*q*_*t*_/*q*_*e*_)) against time (Fig. 3f) nearly passes through the origin with an intercept of approximately zero, demonstrating that film diffusion plays a significant role in Cu^2+^ ion biosorption onto the surface of *R. damascena* leaves. The second part describes the additional Cu^2+^ ion biosorption on the internal pores of the *R. damascena* leaf surface, where intra-particle diffusion is the rate-limiting stage^43^. This result revealed that external diffusion in the film controls the biosorption of copper ions onto *R. damascena* biomass, followed by the intra-particle diffusion model. The high value of the intercept (*C*_*i*_, 6.25 mg/g; Table 4) might be due to increased boundary layer thickness, increased internal mass transfer, and reduced external mass transfer^30^.

### Equilibrium isotherms

Under certain experimental conditions, the adsorption isotherm represents the equilibrium correlation between the amounts of ions adsorbed by the biosorbent and the metal ion concentration in the solution^44^.

Biosorption equilibrium isotherms were obtained under optimized conditions by BBD. The Langmuir, Freundlich, Temkin, Dubinin–Radushkevich and Jovanovic isotherm models were used to describe and estimate the experimental data of copper biosorption.

### Langmuir model

This model implies that metal ions are adsorbed by monolayer adsorption on a homogeneous surface with no interaction between the adsorbed metal ions. The Langmuir model is represented in linear form as follows:

8 $$\frac{{C}_{eq}}{{q}_{e}}=\frac{1}{{q}_{max}b}+\frac{{C}_{eq}}{{q}_{max}},$$

where *q*_*max*_ is the maximal sorption quantity (mg/g) required to produce full monolayer coverage on *R. damascena’s* surface at a high ion equilibrium concentration (*C*_*eq*_; mg/L) and *b* is the constant of the Langmuir model, which is associated with binding site affinity^45^.

The high value of *R*^2^ (0.979; Table 5) indicates that the Langmuir model suitably describes the biosorption process, which is based on the homogeneous distribution of active sites on the surface of the *R. damascena* leaf.

**Table 5:** Isotherm model parameters of the biosorption of Cu^2+^ ions onto *R. damascena*.

Isotherms	Parameters	Values
Langmuir	*q*_*max*_ (mg/g)	25.13
	*b* (L/mg)	0.095
	*R*_*L*_	0.062–0.201
	*R*^2^	0.979
Freundlich	*1/n*	0.313
	*K*_*f*_ (L/mg)	5.98
	*R*^2^	0.735
Temkin	*A* (L/mg)	1.9
	*b *(J/mol)	551.4
	*R*^2^	0.807
Dubinin–Radushkevich	*q*_*o*_ (mg/g)	21.1
	*β* × 10^–6^(mol^2^/J^2^)	6.0
	*E* (KJ/mol)	9.13
	*R*^2^	0.926
Jovanovic	*q*_*max*_ (mg/g)	11.17
	*K*_*J*_ (L/g)	0.01
	*R*^2^	0.589

**Figure 4:**
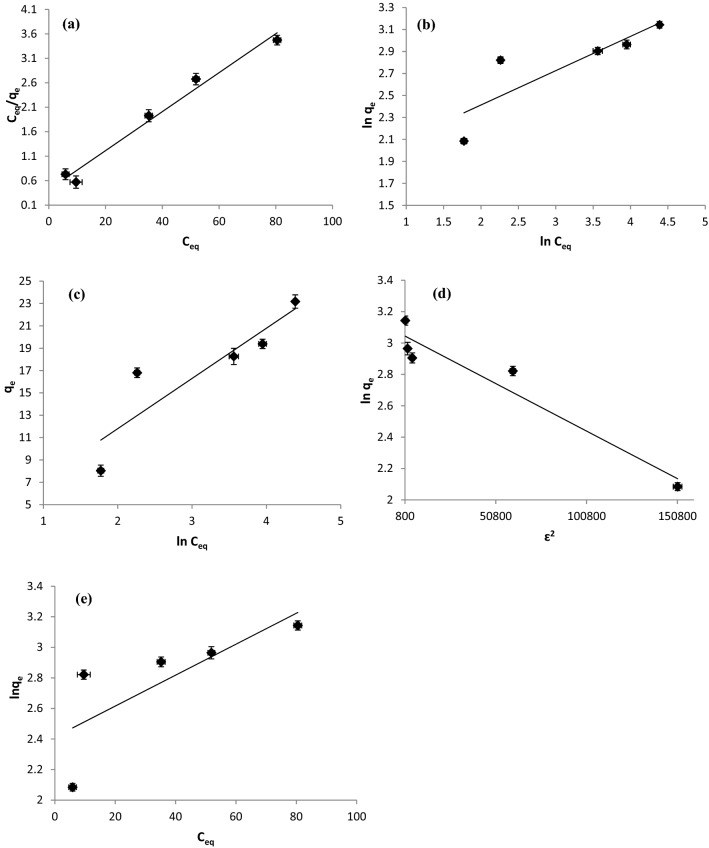
Biosorption isotherms of copper ions onto *R. damascena* leaves, including (**a**) Langmuir, (**b**) Freundlich, (**c**) Temkin, (**d**) Dubinin–Radushkevich and (**e**) Jovanovic models. Data is presented as average ± SE of three replicates.

When *C*_*eq*_*/q*_*e*_ was plotted versus *C*_*eq*_, a straight line was formed, and the slope and intercept were used to determine the *q*_*max*_ and *b* values, respectively (Fig. 4a). The greater value of the Langmuir constant (*b* = 0.095 L/mg) suggested a stronger interaction with the functional groups on the *R. damascena* leaf surface. Furthermore, *R. damascena* biomass had a maximum biosorption capacity (*q*_*max*_) of 25.13 mg/g. A similar pattern was achieved by using numerous equilibrium isotherm models, with the Langmuir model having the best fit^34,46^.

A dimensionless separation factor (*R*_*L*_) may be used to determine the shape and favorability of the biosorption process, which can be computed using Eq. (9):

9 $${R}_{L}=\frac{1}{1+b{C}_{0}},$$

where *C*_o_ is the metal ion concentration (mg/L). The type of Langmuir isotherm was determined by the *R*_*L*_ value, which was either unfavorable (*R*_*L*_ > 1), linear (*R*_*L*_ = 1), irreversible (*R*_*L*_ = 0) or favorable (0 ˂ *R*_*L*_ ˂ 1)^40^. A value of *R*_*L*_ between 0 and 1 indicates that adsorption is favorable. In the current study, the *R*_*L*_ value was determined to be 0.062–0.201, showing that copper biosorption onto the leaves of *R. damascena* is favorable (Table 5).

### Freundlich isotherm model

The adsorption of ions on an energetically heterogeneous surface is described by the Freundlich isotherm model. The following equation represents the linearized Freundlich model (10):

10 $$\text{ln} {q}_{e}=\text{ln} {K}_{f}+\frac{1}{n} \text{ln}{ C}_{eq},$$

where *K*_*f*_ is the Freundlich isotherm constant, which reflects the sorption capacity, and *n* is the Freundlich constant correlated to the adsorption intensity.

The intercept and slope of the plotting of *ln q*_*e*_ against *lnC*_*eq*_ are used to calculate the *K*_*f*_ and *1/n* values, respectively (Fig. 4b). The greater the *K*_*f*_ value, the more biosorbent may be loaded. In addition, adsorption is favorable when the *1/n* value is between 0.1 and 1.0^47^. In this study, the value of *1/n* was lower than 1.0 (0.313; Table 5), indicating that biosorption of copper ions by *R. damascena* leaves is favorable. The low value of the determination coefficient (*R*^2^ = 0.735) suggested that the Freundlich model is not appropriate for describing the experimental data of the biosorption process (Table 5).

### Temkin model

The Temkin model represents adsorption with a uniform distribution of binding energies up to the maximal binding energy, as shown in the following equation^48^.

11 $${q}_{e}= B\text{ln}A+B \text{ln}{C}_{eq},$$

12 $$B=\frac{RT}{b},$$

where *A* (L/mg) represents the equilibrium binding constant, *b* (J/mol) is the constant of the Temkin isotherm model, and *B* (J/mol) is the heat of sorption constant.

The Temkin model constants (*A* and *b*) were determined using the intercept and slope of the *q*_*e*_ versus ln*C*_*eq*_ plot (Fig. 4c). The high *b* value (551.4 J/mol; Table 5) indicates that the adsorbate and biosorbent surface interact strongly^49^. The Temkin model fails to fit the results reported for copper biosorption by *R. damascena* leaves, as the *R*^*2*^ value was low (0.807; Table 5).

### Dubinin–Radushkevich model (D–R)

The D–R model describes whether biosorption occurs via a chemical or physical process and the mean sorption energy of the process. The D–R model is calculated from the following equations:

13 $$\text{ln}{q}_{e}=\text{ln}{q}_{0}-\beta {\varepsilon }^{2},$$

14 $$\varepsilon =RT\left(1+\frac{1}{{C}_{eq}}\right),$$

15 $$E=\sqrt{1/2}\beta ,$$

where *q*_*o*_ is the theoretical maximum capacity (mg/g), *β* is the constant of the D–R model associated with the mean free energy (mol^2^/J^2^), *ε* is the Polanyi potential, *T* (K) is the absolute temperature, *R* (8.314 J/mol K) is the gas constant, and *E* (kJ/mol) is the mean adsorption energy.

Table 5 shows the values of the D–R model parameters. The mean adsorption energy of the system (*E*) was determined using the parameter *β* (Eq. (15)). In addition, the chemical and physical characteristics of the adsorption process may be assessed by the mean adsorption energy.

Physical sorption is defined as a value of *E* less than 8 kJ/mol, whereas chemical sorption is defined as a value of 8 to 16 kJ/mol^30^. The value of *E* (9.13 kJ/mol) indicates that *R. damascena* removed copper ions mostly by chemisorption. This result is also consistent with predictions from the pseudo-second-order and Elovich kinetic models. D–R isotherm model may best describe the experimental data of copper ion biosorption onto the *R. damascena* leaf surface, according to the *R*^2^ value (0.926; Fig. 4d; Table 5).

### Jovanovic model

The Jovanovic model is an approximation for localized monolayer adsorption without lateral contacts that is comparable to the Langmuir model. This model is determined as follows:

16 $$\text{ln}{q}_{e}=\text{ln}{q}_{max}{-{K}_{J}C}_{eq },$$

where *K*_*J*_: the Jovanovic isotherm constant. The values of *q*_*max*_ and *K*_*J*_ were calculated from the intercept and slope of linear plot of *lnq*_*e*_ versus *C*_*eq*_ (Fig. 4e).

The maximum biosorption capacity determined from the Jovanovic equation (*q*_*max*_ = 11.17 mg/g; Table 5) differs from the experimentally measured value (*q*_*max*_ = 23.18). Furthermore, the lower determination coefficient (*R*^2^ = 0.589) found in this investigation revealed that there is a lateral interaction and no mechanical contact between the *R. damascena* leaf biomass and Cu^2+^ ions. As a result, Jovanovic isotherm model has a lower approach to saturation compared to Langmuir model as stated by Al-Ghouti and Da’ana^50^.

### Thermodynamic studies

The Gibbs free energy (*∆G*), enthalpy (*∆H*) and entropy (*∆S*) are all thermodynamic parameters that describe the spontaneity of a biphasic adsorption process^51^.

The following equations demonstrate the relationship between the thermodynamic parameters and the absolute temperature (*T*)^52^.

17 $$\Delta G=\Delta H-T\Delta S,$$

18 $$\Delta G=- RT\text{ln}{K}_{C},$$

19 $${LnK}_{C}=\frac{\Delta S}{R}- \frac{\Delta H}{RT},$$

where *Kc* is the thermodynamic equilibrium constant.

At the experimental temperatures, the values of *∆G* were negative (Table 6), indicating that biosorption was feasible and spontaneous^50^. Furthermore, a reduction in the values of *∆G* with rising temperature indicates that adsorption became more feasible, resulting in the strengthening of bonds established between the binding sites on the *R. damascena* leaf surface and the Cu^2+^ ions^19^.

**Table 6:** Thermodynamic values of Cu^2+^ ion biosorption onto *R. damascena*.

Temperature (K)	Δ*G* (KJ/mol)	Δ*H* (KJ/mol)	Δ*S* (KJ/mol)	*R*^2^
298	− 1.39	21.7	0.077	0.982
308	− 2.00			
318	− 2.94			

**Figure 5:**
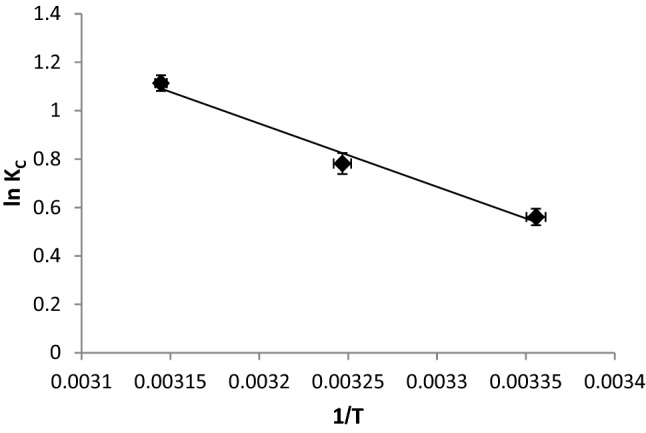
Plot of *1/T* against ln*K* for copper ion biosorption onto *R. damascena*.

The changes in enthalpy and entropy were evaluated from the slope and intercept of the *ln K* versus *1/T* plot, respectively (Fig. 5).

The positive ∆*H* of 21.7 kJ/mol for copper biosorption by *R. damascena* leaves indicates that the biosorption process was endothermic. This result indicates that higher temperatures promote biosorption. In addition, the positive *∆S* indicated that the biosorption of copper ions onto the *R. damascena* leaf surface occurred as a result of randomization at the adsorbate-biosorbent interface^53^.

### Characterization of *R. damascena* leaf surface

#### Scanning electron microscopy (SEM)

**Figure 6:**
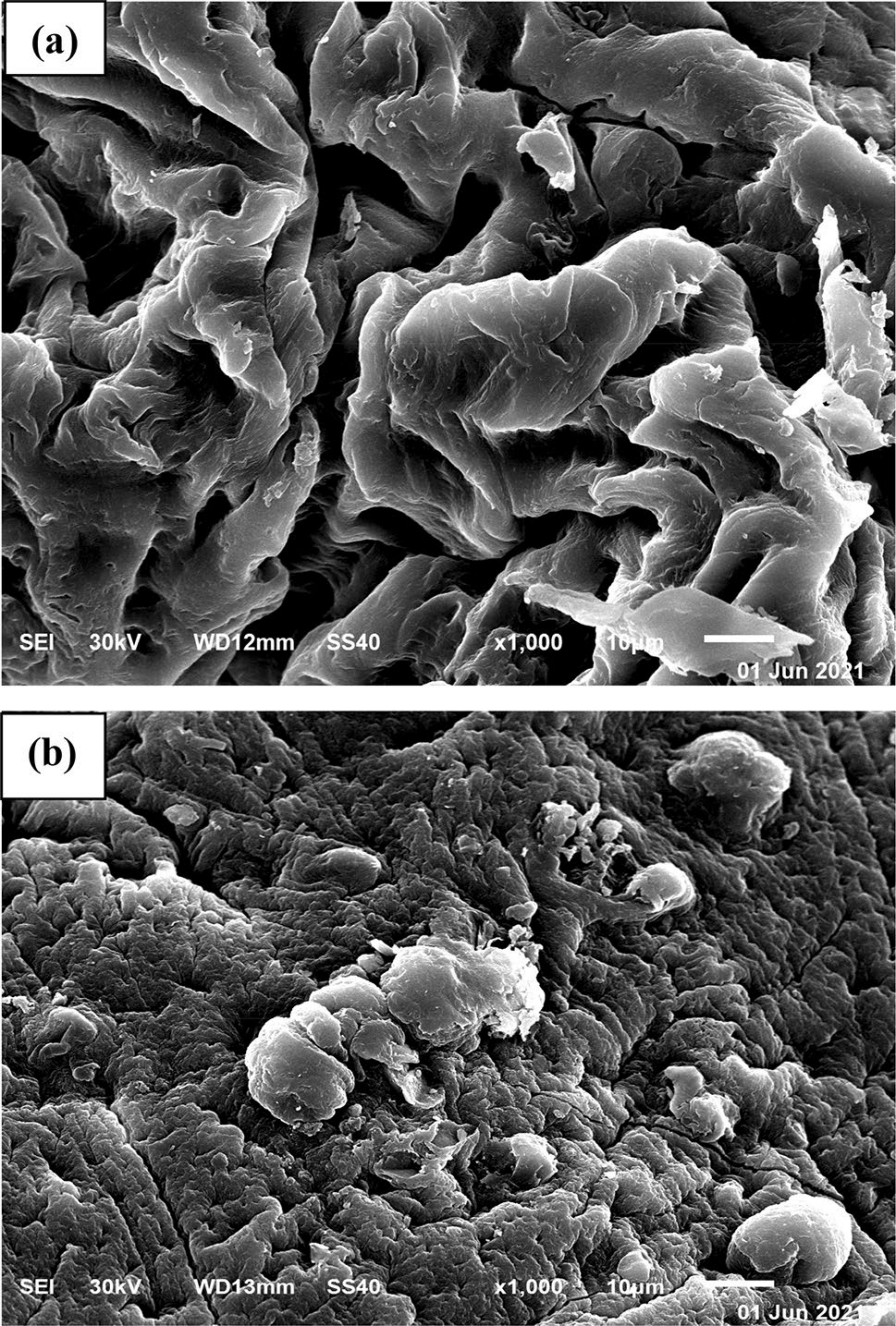
SEM images of *R. damascena* leaves (**a**) before and (**b**) after Cd^2+^ biosorption.

The morphology of the *R. damascena* leaf surface before and after Cu^2+^ biosorption was examined by SEM (Fig. 6). SEM micrographs demonstrated that the surface morphology of the *R. damascena* leaf before and after Cu^2+^ biosorption was different.

The SEM micrographs of the *R. damascena* leaf surface before copper biosorption revealed a rough surface with substantial porosity (Fig. 6a). This rough flaky surface allowed copper ions to adhere more easily, improving biosorption. The biosorbent's porosity also enables it to interact with the adsorbate more quickly^54^. However, the SEM images collected after the biosorption of copper revealed a flatter biosorbent surface, appearance of discrete lumps and fewer large spaces (Fig. 6b). These morphological alterations verified the interaction of copper ions with the functional groups on the *R. damascena* leaf surface^20^.

#### Energy dispersive X-ray spectroscopy (EDX)

**Figure 7:**
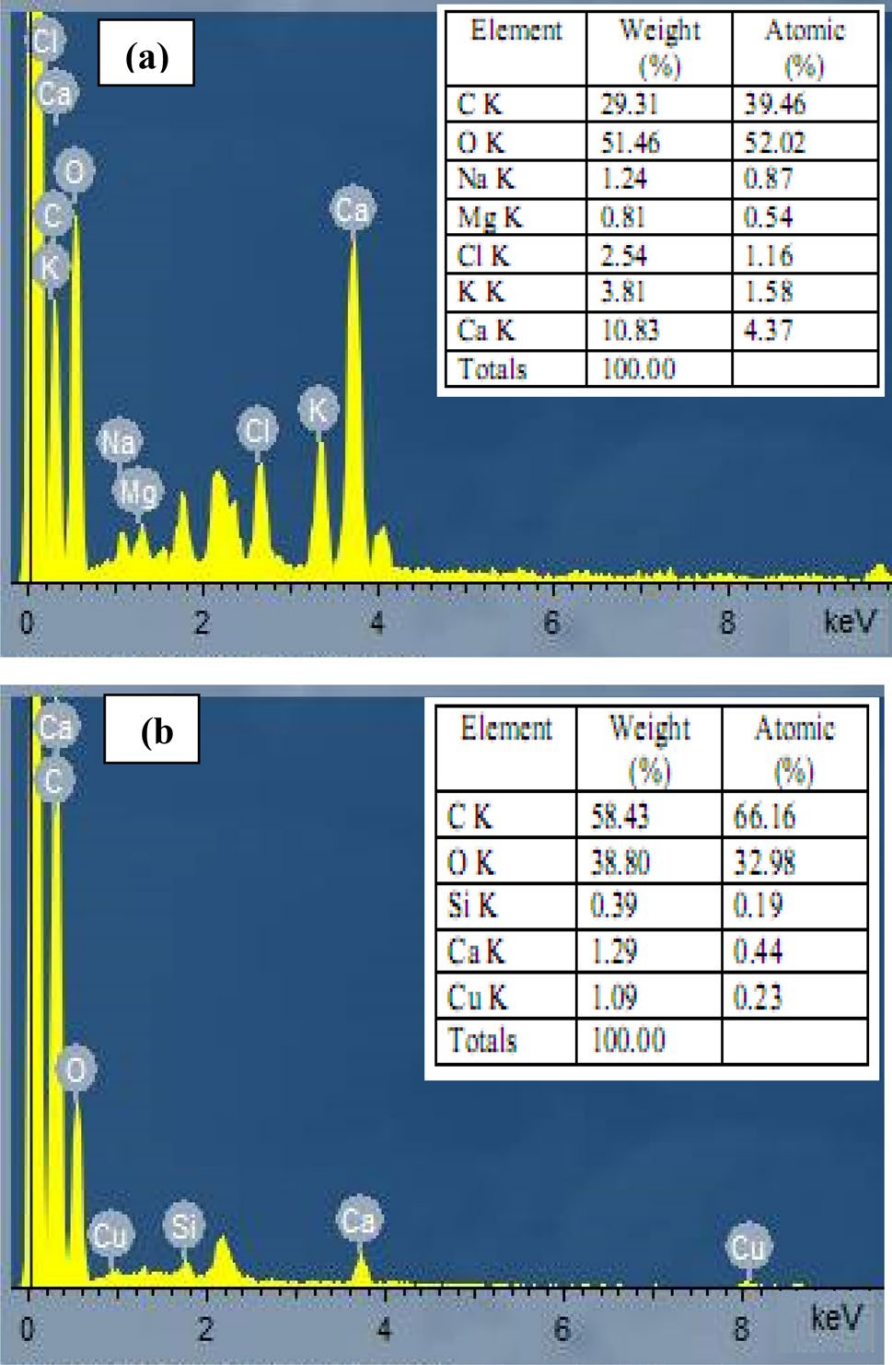
EDX images of *R. damascena* leaves (**a**) before and (**b**) after Cd^2+^ biosorption.

EDX analysis was used to determine the adsorbent surface composition and to confirm the presence of copper ions on the *R. damascena* leaf surface. Figure 7 displays the EDX spectra of *R. damascena* biomass. The EDX spectra showed that the *R. damascena* leaves consist mostly of C and O, with traces of additional elements, including Na, Mg, Cl, K, Si and Ca that were exchanged or removed during biosorption (Fig. 7a,b). This result shows that the biosorption of Cu^2+^ ions was caused by ion exchange. After biosorption, the EDX spectra of *R. damascena* biomass exhibited an additional Cu^2+^ peak (1.09%) on the *R. damascena* leaf surface, demonstrating that the biomass of *R. damascena* participates in the biosorption of Cu^2+^ ions from solution (Fig. 7b). In this regard, El-Naggar et al.^55^ observed that a distinctive copper peak appeared following contact with copper.

### Analysis of the Fourier transform infrared spectra (FT-IR)

**Figure 8:**
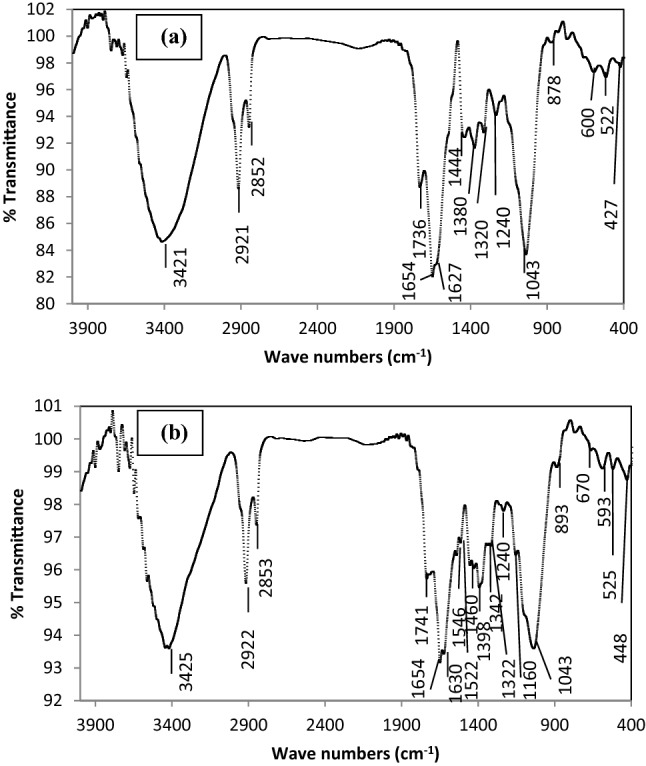
FTIR spectra of *R. damascena* leaves (**a**) before and (**b**) after Cd^2+^ biosorption.

The functional groups found on the surface of biosorbent biomass play a significant role in the process of adsorption. Heavy metal biosorption has been related to various functional groups, such as sulfonate, sulfhydryl, amine, carboxyl, hydroxyl, carbonyl, and others^56^. Figure 8a,b displays the FT-IR spectra of *R. damascena* leaves before and after copper biosorption.

The presence of a wide absorption peak at approximately 3421–3425 cm^−1^ is allocated to O–H stretching of hydroxyl radicals of polysaccharides or water^57^ and to N–H stretching of proteins (amide A)^58^. Functional groups such as O–H and N–H are commonly present in natural cellulose and proteins found in plant cell walls^59^. The O–H stretching vibration of the carboxylic acid might be represented by the bands at 2921 cm^−1^ and 2922 cm^−1^^59^. These bands indicate the presence of an acidic group, such as –COOH, in the biosorbent cell wall; this group serves as a hyperchemical group for the adsorption of various multivalent metal ions^60^. The absorption bands at 2852 cm^−1^ and 2853 cm^−1^ are attributed to stretching of C–H, more specifically to the C–H stretching vibrations of lipids^61^. The C=O stretching of amide I, which is related to proteins, is shown by the absorption peak at approximately 1654 cm^−1^^62^. The appearance of new absorption bands at 1546 cm^−1^ and 1460 cm^−1^ after copper was biosorbed onto the surface of the *R. damascena* leaf might be due to C=O stretching vibrations of different carboxylic and amide (I, II) groups, respectively^63^. The protein band spectrum identified at 1240 cm^−1^ on the leaf surface was caused by the P=O asymmetric stretching vibration^64^. The absorption peak at approximately 1160 cm^−1^ detected only on the *R. damascena* leaf surface following copper biosorption is related to C–O–C stretching of polysaccharides from carbohydrates^57^. Furthermore, after copper biosorption, the peak at 878 cm^−1^ shifted to 893 cm^−1^, indicating the binding of copper ions to the amine group on the leaf surface. The bands found only at 670 and 593 cm^−1^ on the *R. damascena* leaf surface after copper biosorption may be associated with the compounds of organic halide^56^. From Fig. 7, it can be observed that the *R. damascena* leaf biomass included several functional chemical groups, such as carbonyl groups, acids, phosphates, amides, hydroxyl groups, halides, carboxyl groups, and amine groups. They might compensate for the biosorption of copper ions from the aqueous solution onto the *R. damascena* leaf surface.

#### Copper removal by immobilized *R. damascena* biomass

**Figure 9:**
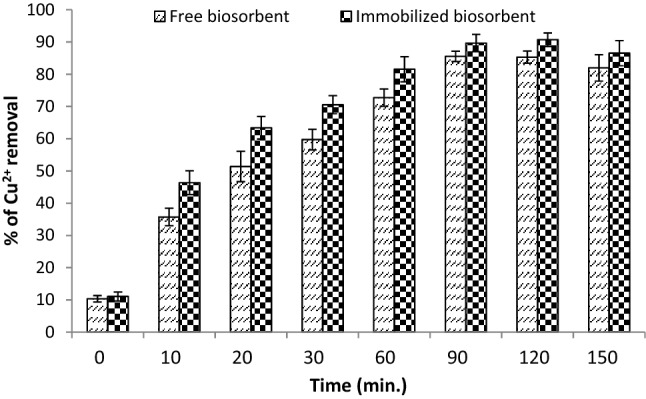
Copper ion removal by Ca–alginate-immobilized *R. damascena*.

The results in Fig. 9 show that the Ca-alginate-immobilized *R. damascena* leaves removed 90.7% of copper ions after 120 min under the conditions optimized by BBD, including the biosorbent dose (4 g/L), pH (5.5) and initial copper concentration (55 mg/L); this removal was higher than the removal achieved when a nonimmobilized biosorbent was used (85.3%). Various studies have found that immobilized biosorbents are a more straightforward approach for recovering and removing heavy metals from wastewater than free biosorbents^65,66^. For example, Ansari et al.^15^ reported that immobilized rose waste is more effective at absorbing Pb^2+^ from aqueous solutions than free biomass. Ca-alginate-immobilized *Fucus vesiculosus* is also an effective biosorbent for copper, lead, and cadmium according to Mata et al.^67^, and it occasionally has greater biosorption efficacy than free alga or even alginate alone. According to Davis et al.^68^, the metal ion affinity for alginate is proportional to the quantity of guluronic acid and other uronic acids present. These acids are responsible for the biosorption of heavy metals since they include the majority of the carboxyl groups in alginate. Furthermore, the “egg-box” structure of the gels, as well as the crosslinking between the carboxyl groups and metal ions, have been linked to alginate’s metal selectivity. This selectivity is determined by the stereochemical environment created by the structure of the gel. Therefore, *R. damascena* immobilized in Ca-alginate has great potential to adsorb heavy metals from wastewater.

### Mechanisms of biosorption

The mechanisms of biosorption for heavy metals include surface precipitation, chelation, complexation, ion exchange, electrostatic interaction, or a combination of these mechanisms depending on the biosorbent used and the conditions of solution^69^. Ion exchange was suggested as a main mechanism for copper ion biosorption onto the *R. damascena* biomass^70^. Light metal ions such as Ca^2+^, Mg^2+^, Na^+^ and K^+^ were described to be involved in the process of ion exchange owing to a poor connection with the biomass of *R. damascena* in comparison to the heavy metals^70^. Moreover, functional groups containing oxygen and/or nitrogen, such as COOH, OH, and NH_2_, help biosorb Cu^2+^ ions by forming hydrogen bonds between the surface of *R. damascena* biomass and Cu^2+^ ions. These findings were supported by the FT-IR analysis because of the shift in the wavenumbers of the COOH, OH, and NH_2_ groups following Cu^2+^ ion biosorption (Fig. 8a,b). The intermolecular hydrogen bonding between the biomass of *R. damascena* and Cu^2+^ ions enhances the biosorption process. The formation of complexes with functional groups on the biosorbent through electrostatic interactions and ion exchange is also a possible mechanism for the biosorption of Cu^2+^ on *R. damascena* biomass.

SEM and EDX analyses were obtained after adsorption to acquire a better understanding of the Cu^2+^ biosorption mechanism by the biomass of *R. damascena*. The SEM analysis displays that the biomass of *R. damascena* is porous with numerous rough pores. The biosorption of Cu^2+^ takes place in the pores of the *R. damascena* biomass. In a comparison of the EDX analyses of *R. damascena* before and after Cu^2+^ ion biosorption (Fig. 7a,b), significant alterations were found along with the appearance of an additional Cu^2+^ peak, indicating that the *R. damascena* biomass was transformed after adsorption. As a result, all of these findings suggest that the biosorption of Cu^2+^ onto *R. damascena* biomass can be accomplished by ion exchange and hydrogen bond formation mechanisms.

### Comparison of biosorption capacity

The maximum biosorption capacity of Cu^2+^ ions by various biosorbents was compared with that observed in the current investigation. Table 7 shows that *R. damascena* leaves have a higher biosorption capacity for copper removal than most of the biosorbents previously described in the literature^20,34,71–77^. The wide availability of *Rosa damascena* leaf wastes and their low cost are added advantages for their selection by numerous industries.

**Table 7:** Maximum biosorption capacity of Cu^2+^ ion by low-cost sorbents.

Biosorbent used for Cu(II)	*q*_*max*_ (mg/g)	References
*Codium vermilara*	14.4	^20^
Bael flowers	23.14	^34^
Sago waste	12.4	^71^
*Myriophyllum spicatum*	10.37	^72^
Wheat shell	10.84	^73^
*Caulerpa lentillifera*	5.57	^74^
Lentil shell	8.98	^75^
*Cinnamomum camphora*	16.76	^76^
Coconut shell	19.89	^77^
Neem leaves	17.49	^77^
*Rosa damascena* leaves	25.13	Present study

## Conclusions

Heavy metals, such as copper, are present in high concentrations in certain industrial effluents, posing serious health and environmental risks. Biosorption is a biotechnological approach to heavy metal ion removal from contaminated aquatic environments. The aim of the current investigation was to optimize the process variables for maximal copper removal from aqueous solution using statistical design. The Box–Behnken experimental design combined with response surface methodology has been shown to be an effective method for maximizing the removal of copper ions from solution using *R. damascena* leaves because they require a decreased number of experimental tests, result in the most efficient conditions, and maintain the accuracy of the predicted response. ANOVA, with its low *P* value, high *F* value, and determination coefficient, showed that the developed model represents the experimental data with high accuracy. The maximum removal percentage of Cu^2+^ ions (88.7%) was reached under the optimal conditions of a biosorbent dose of 4.0 g/L, pH of 5.5, and initial copper content of 55 mg/L. The pseudo-second-order and Elovich kinetic models were best fit to the experimental data. In addition, the liquid film diffusion model initially describes copper biosorption onto the *R. damascena* surface, followed by the intra-particle diffusion model. Equilibrium isotherm studies demonstrated that the Langmuir and D–R isotherm models could describe Cu^2+^ biosorption better than the Freundlich, Temkin and Jovanovic models, with the highest monolayer biosorption capacity of 25.13 mg/g, suggesting chemical interactions between the metal ions and biosorbent. Thermodynamic parameters such as Gibbs free energy, enthalpy, and entropy showed that the biosorption process is spontaneous, feasible and endothermic. After biosorption, SEM and EDX spectroscopy indicated noticeable alterations in the properties of the *R. damascena* leaf surface. In addition, FT-IR spectroscopy revealed the existence of functional groups, such as carbonyl groups, acids, phosphates, amides, hydroxyl groups, halides, carboxyl groups, and amine groups, in the *R. damascena* leaf biomass, all of which are likely to be involved in the biosorption of copper ions. Immobilization was shown to be a promising method for producing efficient adsorbents that can be used to sequester metal ions from wastewater. Therefore, *R. damascena* leaves can be used as a low-cost biosorbent to remove copper ions from aqueous solutions.

## Materials and methods

### Preparation of biosorbent

*Rosa damascena* Miller var. *trigintipetala* Dieck was collected from Taif rose farms in the Al-Shafa highland, Taif region, Saudi Arabia. Voucher specimens were deposited and identified by staff members of the herbarium at Taif University, Taif, Saudi Arabia. The rose leaves were removed from the plants, washed under running water to eliminate any impurities or pollutants, and then dried at room temperature for two weeks. Until further investigation, the leaves were crushed into a fine powder and stored in an airtight container.

### Preparation of copper solutions

Approximately 3.93 g of copper sulfate (CuSO_4_·5H_2_O) was dissolved in 1000 mL distilled water to prepare the Cu^2+^ stock solution. All of the chemicals used in this investigation were of analytical grade and obtained from Sigma–Aldrich, including CuSO_4_·5H_2_O, HCl, NaOH, Na-alginate and CaCl_2_.

### Batch biosorption experiments

#### Impact of individual factors

The impact of different variables on biosorption by *R. damascena* leaf biomass was investigated using batch experiments. The effect of the initial Cu^2+^ concentration (30–150 mg/L), temperature (25–45 °C) and contact time (0–150 min) was examined. The biosorption tests were carried out in 250 mL conical flasks with 100 mL of copper solution, and the mixture was agitated at 170 rpm in a shaker. Deionized water was used to prepare the solutions, and the pH was adjusted using 0.1 M HCl or 0.1 M NaOH.

Each experiment was repeated three times, with the average results provided. The biosorbent was removed from the solutions at the end of the biosorption procedure by centrifugation for 5 min at 4000 rpm.

The concentration of copper in the filtrate was determined by inductively coupled plasma–optical emission spectrometry (ICP–OES) (Perkin Elmer Optima 2000 DV). The following equation was used to calculate the biosorption of copper ions onto *R. damascena* leaf biomass (*q*_*e*_; mg/g).

20 $${q}_{e}=\frac{V \left({C}_{i}-{C}_{eq}\right) }{W},$$

where *C*_*i*_ and *C*_*eq*_ (mg/L) are the copper ion concentrations before and after the equilibrium contact time, *V* (mL) is the volume of copper solution, and *W* (g) is the weight of *R. damascena* leaf powder.

The removal percentage of Cu^2+^ ions by *R. damascena* leaves was calculated by Eq. (21):

21 $$\mathrm{Removal }\left(\mathrm{\%}\right)=\frac{\left({C}_{i}-{C}_{eq}\right) }{{C}_{i}}\times 100.$$

### Optimization of Cu^2+^ removal by Box–Behnken statistical design (BBD)

Response surface methodology is a multivariable optimization approach that fits experimental results to a second-order equation to determine the optimal response of a process that is a function of numerous independent factors. Designing an experimental matrix, developing a mathematical model, and optimizing the response are the three key processes of RSM^78^. Experiments were developed to determine the optimal copper biosorption onto *R. damascena* leaves using Box–Behnken Design and RSM (Stat-Ease Inc., Minneapolis, USA). The design included 17 runs with three independent factors, biosorbent dose (1, 3, 5 g/L), pH (2, 4, 6) and initial copper concentration (30, 60, 90 mg/L), at three coded levels (− 1, 0, + 1) (Table 1).

The sorption experiments were carried out with a fixed contact time of 90 min at 25 °C and 180 rpm, and the remaining concentration of Cu^2+^ ions was then determined as previously described.

The following equation is a second-order polynomial equation that includes the independent factors and the dependent response.

22 $$Y \, = \, \beta_{0} \, + \, \sum \, \beta_{i} X_{i} + \, \sum \, \beta_{ii} X_{i}^{2} + \, \sum \, \beta_{ij} X_{i} X_{j} + \varepsilon ,$$

where *Y* is the expected response, *β*_0_ is the intercept term, *β*_*i*_, *β*_*ii*_ and *β*_*ij*_ are the linear, quadratic, and interaction impacts, respectively, *X*_*i*_ and *X*_*j*_ are the independent variables, and *ε* is the error.

The optimized conditions obtained from BBD were used to determine the kinetic biosorption models at various time periods (0–150 min) and to estimate isothermal models at various initial Cu^2+^ concentrations (30, 60, 90, 120 and 150 mg/L) and a contact time of 90 min at 25 °C. Thermodynamic investigations of Cu^2+^ ion biosorption were also examined, with the biosorption process being tested under optimal conditions at different temperatures (25, 35, and 45 °C).

### Validity of kinetics models

The applicability and validity of the biosorption kinetic models was established by determining the normalized standard deviation (NSD) and chi-square (*X*^2^).

The lower the NSD and *X*^2^ values for a given kinetic model are, the more likely the experimental data are to be valid^79^.

The mathematical equations of NSD and *X*^2^ are given as follows:

23 $$\Delta {q}_{e}\left(\%\right)=100\sqrt{\left[\frac{\left({q}_{exp.}-{q}_{cal.}\right)/{q}_{exp.}}{N-1}\right],}$$

24 $${X}^{2 }=\sum_{i=1}^{n}\frac{{\left({q}_{exp.}-{q}_{cal.}\right)}^{2 }}{{q}_{cal.}},$$

where *N* is the number of data points.

### Statistical analysis of the data

It has been stated that three-dimensional response surface plots can be used to analyze the principal and interaction impacts of two parameters while all other variables are held constant. The regression model was used to produce the 3D response surface plots for the copper removal percentage by keeping one factor at the center level. The experimental design and statistical analysis were performed in Design Expert version 7. Multiple regression analysis and ANOVA were used to assess the experimental data, and significance was determined at probability levels using the *F* test (*p* ≤ 0.05). The Duncan’s multiple range tests were performed to compare the means using the SPSS statistical package (version 16.0).

### Characterization of *R. damascena* leaves

A scanning electron microscope (SEM-JEOL JSM-6510 L. V operated at 30 kV) with energy-dispersive X-ray spectroscopy (EDX, JEOL JEM-2100 (HRTEM) operated at a voltage of 200 kV) was used to examine the surface morphology of the *R. damascena* leaves. The functional groups on the *R. damascena* leaf surface were determined using Fourier transform infrared radiation spectroscopy (FTIR, Thermo Fisher Scientific model FT-IR is 10, USA) before and after biosorption process.

### Biosorbent immobilization

*Rosa damascena* leaves were immobilized for 30 min at 60 °C under continuous stirring by the dissolution of 4 g of Na-alginate in 100 mL distilled water^80^. In the Na-alginate solution, 4 g/L *R. damascena* leaf powder was added. This mixture was then added to a 2% CaCl_2_ solution using a 3 mL syringe to form beads. For full gelation, the spherical beads (3 mm) were maintained for 2 h in a 2% calcium chloride solution. The beads were then rinsed in distilled water to eliminate any excess CaCl_2_ and stored in the refrigerator until they were needed again. The biosorption experiment was performed as previously described using immobilized *R. damascena* leaves and non-immobilized biosorbent as a control under the optimum conditions obtained by BBD at a temperature of 25 °C and over various time intervals (0–150 min).

All methods were performed in accordance with relevant guidelines and regulations.
